# Molecular Studies on Cystic Echinococcosis of Camel (*Camelus dromedarius*) and Report of *Echinococcus ortleppi* in Iran

**Published:** 2017

**Authors:** Mohammad EBRAHIMIPOUR, Seyed Mahmoud SADJJADI, Hossein YOUSOFI DARANI, Mohsen NAJJARI

**Affiliations:** 1. Dept. of Parasitology and Mycology, School of Medicine, Shiraz University of Medical Sciences, Shiraz, Iran; 2. Basic Sciences in Infectious Diseases Research Center, Shiraz University of Medical Sciences, Shiraz, Iran; 3. Dept. of Parasitology and Mycology, School of Medicine, Isfahan University of Medical Sciences, Isfahan, Iran

**Keywords:** *Echinococcus ortleppi*, Mitochondrial DNA genes, Phylogenetic analysis, Camel, Iran

## Abstract

**Background:**

Cystic echinococcosis (CE) is one of the most important zoonotic diseases; caused by different genotypes of *Echinococcus* spp. Camels have an important role in transmission cycle of *E. granulosus* especially, in desert areas. This study aimed to investigate molecular characterization of hydatid cysts isolates from one-humped camel (*Camelus dromedarius)* and to show its molecular and phylogenic status in this important CE host in the central part of Iran.

**Methods:**

Twenty hydatid cyst samples (14 fertile and 6 calcified) were collected from 56 slaughtered camels in Central part of Iran. Extraction of DNA from 14 fertile samples was achieved followed by molecular studies on two mitochondrial genes (*nad1* and *cox1*).

**Results:**

Blast and phylogenetic analysis on sequenced genes showed the presence of G1 (28.6%), G3 (28.6%) and G6 (35.7%) genotypes in the samples. However, one sample was detected as *E. ortleppi* (G5) with 99% homology with G5 isolated from camel in Egypt (AB921055) and Sudan (JX912709).

**Conclusion:**

Presence of *E. ortleppi*, originally the cattle genotype, is reported for the first time in Iran. Due to the potential of infecting human by *E. ortleppi*; more attention should be paid to this zoonotic genotype in this region.

## Introduction

Hydatidosis/cystic echinococcosis (CE), an important zoonotic helminthic disease, remains as a health problem with a large socioeconomic burden in many parts of the world including the Middle East (1–3). As an endemic area for CE, the disease is responsible for about 1% of surgical admissions in Iran (4).

Molecular studies based on nuclear and mitochondrial genomes have showed the *Echinococcus granulosus* as G1 to G10 strains (Sensu lato), *E. granulosus* sensu stricto (strain G1–G3), *E. equinus* (G4), *E. ortleppi* (G5) and *E. canadensis* (G6–G10) that has recently been named as *E. intermedius* (5–8). Hydatid cysts are usually found in sheep, camel, cattle and goat throughout Iran. The G1, G2, G3, G6 and recently G7 genotypes have been reported from Iran, so far (9–18). Camels have an important role in transmission cycle of the parasite and usually are infected with G6 genotype of *E. granulosus* (19). Three genotypes of *E. granulosus* including G6 (camel strain) with higher prevalence, G1 (sheep strain) and G3 (buffalo strain) have been reported from camels in Iran (20–25). To date, only camel strain (G6 genotype), sheep strain (G1–G2 genotypes), and buffalo strain (G3) have been detected from human in Iran (26–28). The proper environmental and ecological condition, emigrant population, none industrial abattoirs, home slaughtering, and large number of stray dogs are the major factors of distributing the disease in endemic countries (29–31).

*E. ortleppi* (G5 strain) as a common cattle strain that is geographically distributed in Europe, Africa, Southern Asia and the Americas (7, 32); has been reported from camel in Sudan and Egypt (33, 34). Human infection by this genotype has also been reported from several countries including Argentina, Brazil, Mexico, Netherlands, South Africa, France and India (35–37).

This study aimed to investigate molecular and phylogenetic data on hydatid cysts isolated from one-humped camel (*Camelus dromedarius)* in Iran.; where, G1, G3, and G6 genotypes have been reported from camel, earlier (20, 24).

## Materials and Methods

Twenty hydatid cysts samples including 18 (90%) cysts from lung and 2 (10%) cysts from liver of 56 slaughtered camels were collected from February to March 2015 in Najaf Abad district abattoir, Isfahan Province, Central part of Iran. The samples were immediately transferred to the laboratory in cool condition. Hydatid cyst fluids (HCF) including protoscoleces (PSCs), were collected by sterile syringes and transferred into suitable and clean falcon tubes. The tubes were centrifuged at 3000 × g. The PSCs were collected and transferred into alcohol 70% for molecular studies.

Micro-tubes containing PSCs were centrifuged at 3000 × g followed by removing their supernatants. The packed sediment (30–100 μl) was transferred into a new 1.5 micro-tube and washed three times by distilled water for removing of excess alcohol. A total of 300 μl of lysis buffer was added to each sample. Freeze and thaw procedure was applied to each tube for five times-each for 3 min-using liquid nitrogen and boiling water for surface cracking of PSCs. A total of 25 μl of proteinase K was added to each sample and incubated at 37° C overnight. The DNA was extracted by phenol-chloroform protocol. In brief, 300 μl of phenol-chloroform-isoamyl alcohol was added to each sample and centrifuged at 2000 × g for 5 minutes. The supernatant was transferred into a new micro-tube and previous step was repeated. The same volume of absolute ethanol was added to the supernatant. Sodium acetate 3M, was added as much as 0.1 of the mentioned volume and was incubated at −20° C for 30 minutes. The sample was then centrifuged at 5000 × g for 12 minutes and its supernatant was poured off. The pellet was added a total of 300 μl of ethanol 70 % and centrifuged at 2000 × g for 5 minutes. The supernatant was discarded followed by waiting for drying the remaining alcohol from the samples and finally each sample was added 50 μl of deionized water and transferred into −20° C, until use.

The Cytochrome c oxidase subunit 1 (*cox1*) and NADH dehydrogenase subunit 1 (*nad1*) genes were amplified by two primers as follows: JB3 (5′-TTTTTTGGGCATCCTGAGGTTTAT-3′) and JB4.5 (5′-TAAAGAAAGAACATAATGAAAATG-3′) for *cox1* gene and JB11 (5′-AGATTCGTAAGGGGCCTAATA-3′) and JB12 (5′-ACCACTAACTAATTCACTTTC-3′) for *nad1* gene as forward and reverse primers, respectively (38, 39).

PCR reagents and thermal cycler program were similar in both *cox1* and *nad1* genes amplification. PCR reactions were applied in a final volume of 50 μl, including 2.5 μl genomic DNA, 3.5 mM MgCl2, 250 μM of dNTPs, 25 p mol. of each primer and 2 U of Taq polymerase. The following temperature profile was used for DNA amplification: 40 cycles of 94° C for 45 s, 51° C for 35 s, 72° C for 45 s, followed by a final extension at 72° C for 10 min. Positive (confirmed DNA samples) and negative (no added DNA) controls were used for each PCR program for accuracy. PCR products were visualized using electrophoresis with 1.5 % agarose gel in TAE buffer and stained with GelRed (Biotium®). A 100-bp molecular ladder was used as DNA size marker in each gel for estimating the size of the bands. Gels were observed and photographed using a UV-trans illuminator (Uvitec®).

All PCR primary products of both *cox1* and *nad1* genes were purified by purification kit (Vivantis®) and sequenced in two directions using the similar forward and reverse primers applied in the PCR. Sequence results were edited and aligned by *Genius* (40) and *BioEdit* (41) softwares.

To confirm the identity of the obtained sequences in comparison with the GenBank nucleotide database, all samples were blasted using NCBI (National Center for Biotechnology Information, Bethesda, MD, USA). Phylogenetic trees were constructed using Maximum Likelihood Tree implemented in MEGA software version 7 (42). Bootstrap analysis was used to evaluate the reliability of inferred trees from MEGA 7 software. Nucleotide sequences of *cox 1* and *nad1* genes belonged to *Taenia saginata* with GenBank accession numbers AB494480 and AM503345, were used as out groups in the phylogenetic trees, respectively.

## Results

Out of 20 hydatid cyst samples, 18 (90%) cysts belonged to lung and two (10%) to the liver. A total of 70% (14/20) of the cysts were fertile while, 30% (6/20) were calcified. DNA isolation from calcified cysts was negative so, they were ruled out from molecular studies. PCR-based assay with specific primers yielded two different bands of 450-bp and 470-bp in PCR of *cox1* (12 samples) and *nad1* (3 samples) genes, respectively. DNA isolation from fourteen fertile cysts was successful which their PCR products were sequenced. Blast analysis of the sequenced data using GenBank database, indicated the presence of G1 in 28.6% (4/14), G3 in 28.6% (4/14), G6 in 35.7% (5/14) and G5 in 7.1% (1/14) isolates in the current study. The amplified genes and the accession numbers for the detected strains are shown in Table 1. It should be considered that detected G5 genotype, using *cox1* gene, subsequently, was also confirmed by amplification of *nad1* gene. The partial sequences generated from *cox1* and *nad1* genes for G5 strain were deposited in the GenBank under the accession numbers KT988115 and KT988119, respectively.

**Table 1:** Information about sequences that used for phylogenetic analysis of cox1 and nad1 genes

**Accession number (cox1)**	**Genotype of *Echinococcus***	**Reference**	**Accession number (nad1)**	**Genotype of *Echinococcus***	**Reference**
KU756222	G1	This study	JN579164	G1	Sadjjadi et al. (2013)
KU756223	G1	This study	JN579165	G1	Sadjjadi et al. (2013)
KU756224	G1	This study	KF731955	G1	Nikmanesh et al. (2014)
KU756225	G1	This study	AB921092	G5	Amer et al. (2015)
KF731903	G1	Nikmanesh et al. (2014)	JN637177	G5	Ahmed et al. (2013)
KT074949	G3	Tanzifi et al. (2015)	AB979274	G5	Morishima et al. (2014)
KT988111	G3	This study	KT988119	G5	This study
KT988112	G3	This study	HM749616	G6	Rostaminejad et al. (2010)
KT988113	G3	This study	KT988120	G6	This study
KT988114	G3	This study	KT988121	G6	This study
JX912709	G5	Ahmed et al. (2013)	AM503345	*Taenia saginata*	Zhang et al. (2007)
KT988115	G5	This study			
AB921055	G5	Amer et al (2015)			
KP751426	G6	Karamian et al. (2015)			
KT988116	G6	This study			
KT988117	G6	This study			
KT988118	G6	This study			
AB494480	*Taenia saginata*	Abe et al. (2009)			

In our G3 and G1 genotypes (obtained by *cox1*) there was only one alteration in nucleotide comparing to the used reference sequences: (KT074949) for G3 and (KF731903) for G1 genotypes, respectively. In five G6 samples detected by *cox1* and *nad1* primers in the present study, 99% homology was observed with reference sequences used in the phylogenetic trees. The G5 genotype isolated from camel in Egypt (AB921055) and Sudan (JX912709) were 99% identical to the G5 detected in the current study with only one-alteration nucleotide sequences. However, G5 of the current study (cox1) had 100% homology with G5 obtained from lemur (KU378107) and spotted deer (JX068638) in the United Kingdom.

**Fig. 1:**
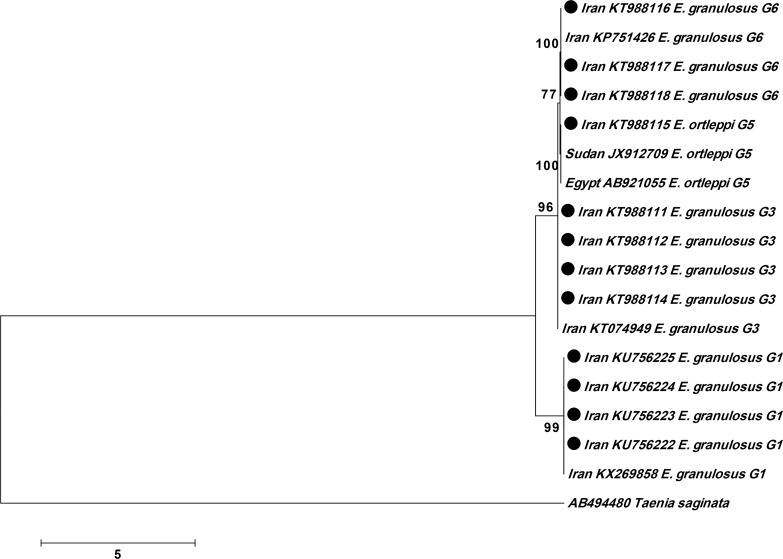
Genetic relationships of obtained genotypes from camel in the present study and reference sequences related genotypes of *E. granulosus* as well as *Taenia saginata* as the out-group. The relationships were inferred based on phylogenetic tree (*cox1* gene). The phylogenetic tree was constructed using Maximum Likelihood Tree implemented in MEGA software version 7

**Fig. 2:**
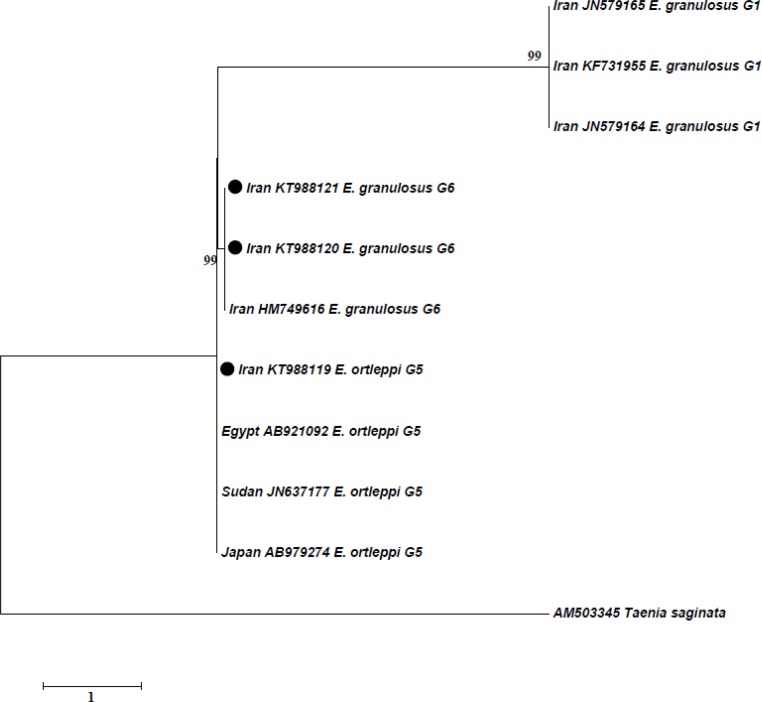
Genetic relationships of obtained genotypes from camel in the present study and reference sequences related genotypes of *E. granulosus* as well as *Taenia saginata* as the outgroup. The relationships were inferred based on phylogenetic tree (*nad1* gene). The phylogenetic tree was constructed using Maximum Likelihood Tree implemented in MEGA software version 7

The relationship between these isolates and other similar genotypes identified worldwide are shown by phylogenetic trees for *cox1* (Fig. 1) and *nad1* (Fig. 2) genes. Using *cox1* and *nad1*, 18 and 12 isolates were analyzed, respectively and related phylogenetic trees were constructed (Fig. 1 and Fig. 2).

## Discussion

Cystic echinococcosis (CE), affects many people throughout the world, although, advances in diagnosis and treatment of CE had been achieved in the recent years. There is still a limit to the disease control. As an endemic region with high incidence of CE, the disease is considered as a public health and socioeconomic problem in Iran, yet (2–4). To control this disease, strategies including surveying on different aspects of parasite should be more considered in the endemic regions (43). However, investigations on the epidemiology and different genotypes of parasites in the intermediate and final hosts should be considered in any endemic area to achieve the evidence-based control and management programs (36).

Different genotypes of *E. granulosus* including G1, G2, G3, G6 and G7 have been reported from different hosts in Iran (9–18). Camels as important intermediate hosts for CE, specially, in desert areas, have been studied in Iran and molecular studies on nuclear and mitochondrial genes have indicated the presence of G6 genotype as the dominant genotype of *E. granulosus* in camels (3, 23, 44, 45); however, the presence of G1 and G3 genotypes in camel hydatid cysts have also been reported from Iran (21, 24, 25).

The G6 genotype has been known as common camel strain but, in some areas, G3 genotype of *E. granulosus* has been shown the dominant genotype in camels (24, 44); however, G1 has also been considered as a noticeable genotype in camels (21, 22, 25). In the current study, G6 was the dominant genotype and detected in 35.7% of samples. The highest infection rate in camel has been reported from Isfahan and Khorasan Razavi and the lowest rate in Kerman and Semnan Provinces (22, 46). In the present study, the lungs were the most infected organ, which is similar to previous studies (9, 46, 47).

In the African countries, the G6 has been reported as the dominant genotype (3, 48). The genotype of all isolates from camel in Mauritania, Algeria and Sudan has been reported to be G6 (49–51). However, other studies in Kenya and Libya have shown a noticeable prevalence of G1 strains in camel isolates (51, 52). The G5 genotype, its host and distribution is different in the world. Investigation of 638 fertile cysts of cattle has shown the presence of G1 (56.6%) and G5 (43.4%) genotypes in Brazil, while the G5 was mostly isolated from lungs (53). The G5 genotype has been isolated from cattle cysts in Argentina and Italy (54, 55) and in spotted deer from UK, too (56).

Existence of G5 strain has already been reported from camel in Sudan and Egypt (33, 34). This genotype has also been reported from human in Argentina, Brazil, Mexico, Netherlands, South Africa, India and France (7, 35, 37), which makes it as an important genotype in the view of public health. The G5 genotype isolated from camel in Egypt (AB921055) and Sudan (JX912709) were 99% identical to the G5 detected in the current study with only one alteration in nucleotide sequences and similar to other studies in G5 genotypes in camel. However, G5 of the current study (*cox1*) had 100% homology with G5 obtained from lemur (KU378107) and spotted deer (JX068638) in the United Kingdom (56).

The camel as a natural intermediate host for *E. granulosus* plays an important role in the maintaining of the parasite in the nature especially in desert areas (43). On the other hand scattered camels in desert and semi desert areas, where other ruminants and carnivorous animals may live in Iran’ could be infected with this important genotype.

## Conclusion

As far as our knowledge, the present study genetically showed the presence *E. ortleppi* (G5) in camel for the first time in Iran. However, other zoonotic genotypes including the G1, G3 and G6, which have been reported from camel, were also detected in the present study. Due to the transmission potential of G5 strain to human, the finding of *E. ortleppi*, in camel should be more noticed in Iran. The distribution of G5 genotype in Iran is not known. However, more studies are needed to find the distribution of G5 genotype in Iran. More molecular studies on cattle and camel hydatid cysts are needed to find the main reservoir of *E. ortleppi* in Iran. Moreover, molecular and parasitological studies on different final hosts will evaluate the probable existence and its circulation in this region.
